# The Effects of Oyster Mushroom Stem Waste Dietary Supplementation on Growth Performance, Meat Quality Parameters, and Antioxidant Capacity in Lambs

**DOI:** 10.3390/ani16162504

**Published:** 2026-08-11

**Authors:** Agori Karageorgou, Ioanna Kostogiannou, Vasileios Petrovas, Stella Triantafillou, Pantelis Mytilinis, Dimitrios Konstantas, Theofilos Massouras, Michael Goliomytis, Ioannis Politis, Panagiotis Simitzis, Ouranios Tzamaloukas

**Affiliations:** 1 Department of Animal Science, Agricultural University of Athens, 11855 Athens, Greece; akarageorgou@aua.gr (A.K.); mgolio@aua.gr (M.G.); i.politis@aua.gr (I.P.); 2 Department of Food Science and Human Nutrition, Agricultural University of Athens, 75 Iera Odos, 11855 Athens, Greecetheomas@aua.gr (T.M.); 3 Department of Agricultural Sciences, Biotechnology and Food Science, Cyprus University of Technology, Limassol P.O. Box 50329, Cyprus; ouranios.tzamaloukas@cut.ac.cy

**Keywords:** *Pleurotus ostreatus*, mushroom residues, sheep, growth characteristics, meat technological and sensory properties, meat oxidative stability, meat fatty acid profile

## Abstract

Oyster mushroom cultivation results in the generation of large amounts of by-products, the improper disposal of which can have adverse environmental consequences. The utilization of mushroom stem waste as a functional feed ingredient could serve as a promising strategy for its sustainable management and the development of alternative feed resources. This experiment investigates the effects of dietary enrichment with dried oyster mushroom stem waste of the *Pleurotus ostreatus* species (OMSW) on lamb growth performance and meat quality indices. Twenty-four 3-month-old male Chios lambs were randomly assigned to three groups; a control group (C) that received a basal diet; the P2 group that was offered a concentrate supplemented with 2% dried OMSW; and the P4 group, supplemented with 4% OMSW. Individual feed intake and body weight measurements were recorded weekly during the 28-day experimental period for each lamb. At the end of the feeding trial, lambs were fasted for 12 h and slaughtered. The internal organs (spleen, kidneys, liver, lungs, and heart) were then weighed, along with the hot carcass for each lamb. After 24 h at refrigerated storage, cold carcass weight was recorded and samples of *longissimus thoracis* muscle were collected from each lamb for subsequent meat quality assessment. No significant differences were observed among the three groups in feed intake and body weight throughout the experiment. Hot and cold carcass and internal organs’ weights were also not affected by OMSW addition. Moreover, meat quality characteristics, such as pH, color, water holding capacity, shear force values and intramuscular fat were similar among the experimental groups. Meat oxidative stability during refrigerated storage was ameliorated (*p* = 0.002), possibly due to the antioxidant activity exerted by the phenolic compounds contained in OMSW, without any adverse effects on the meat fatty acid profile. These findings indicate that OMSW may be successfully incorporated into growing lamb diets up to 4% as an alternative feed without adverse effects on intake, production or meat quality.

## Simple Summary

Mushroom cultivation generates considerable quantities of by-products, many of which remain underutilized or are discarded as waste. Consequently, increasing attention has been directed toward the valorization of mushroom stem waste as sustainable alternative feed resources for livestock production. The objective of this study was to evaluate the effect of dried stems of the oyster mushroom on lamb growth and meat quality. Twenty-four lambs were allocated into three groups; one group was fed a normal diet, and the other two groups were fed diets containing varying levels of mushroom stem waste. Results showed that lambs fed the mushroom waste were comparable in growth to the controls. Meat quality characteristics, such as tenderness and colour, also remained unchanged. Furthermore, dietary inclusion of mushroom by-products improved lamb meat oxidative stability during refrigerated storage, potentially due to the antioxidant activity of naturally occurring bioactive compounds present in the mushroom stem waste. The findings of the present study support the utilization of oyster mushroom stem waste as a sustainable alternative feed ingredient for lamb production, promoting agricultural by-product valorization, improving resource-use efficiency, and potentially enhancing the quality and shelf life of lamb meat.

## 1. Introduction

Agro-industrial waste constitutes a substantial global environmental, economic, and societal concern, as an estimated 10–50% of agricultural products are lost or discarded annually, resulting in considerable resource inefficiencies and environmental impacts [1]. As a result, there has been a significant increase in the global interest in circular economy approaches that improve sustainable food production and lead to effective management of agro-industrial waste [1]. Circular economy models aim to reduce waste and improve resource efficiency by transforming byproducts into valuable raw materials for other systems [2]. A common practice utilized to promote economic growth in alignment with circular economy principles is the cultivation of edible mushrooms. Edible mushrooms, such as *Pleurotus ostreatus* and *Lentinula edodes*, can be grown on various agro-industrial wastes, such as corn stover [3], crop residues, cotton stalks [4], paper scraps, and spent coffee grounds [5]. Thus, mushroom cultivation is sustainable from both an economic and an environmental perspective. Over the next five years, the global mushrooms market is expected to grow at an exponential rate [6].

During oyster mushroom cultivation, great quantities of residues, such as spent mushroom substrate, stems or non-compliant fruiting bodies are produced. Oyster mushroom stems represent 25–33% of mushroom fresh weight and although are considered as a waste by-product [7], they can serve as a cost-effective alternative feed resource for livestock [8]. As indicated in the literature, stems possess similar composition with fruiting bodies apart from a higher content of structural polysaccharides, i.e., chitin, hemicellulose, and complex glucans [9]. At the same time, OMSW is a valuable source of bioactive compounds, namely phenolics, β-glucans, mannogalactans and ergosterol, which possess well-documented antioxidant, immunomodulatory, and health-promoting properties [10]. Therefore, utilization of OMSW in animal diets emerges as a promising option with benefits for both the environment and animal production.

However, although there are few studies on the application of mushroom by-products in lamb diets, these focus primarily on growth performance or on the use of spent mushroom substrate (SMS) as a source of roughage. In general, the inclusion of SMS in lamb diets may enhance animal health and productive performance by modulating the ruminal microbiota and exerting probiotic, antioxidant, and anti-inflammatory effects [11,12]. There are significant differences in the composition (%) of SMS [13] and mushroom stem waste [14] derived from *Pleurotus ostreatus* cultivation; 4.7 vs. 12.6, 26.7 vs. 10.2, 18.4 vs. 7.61 and 1.40 vs. 2.09 for crude protein, crude fiber, ash and ether extract, respectively, indicating a more favorable nutritional composition for OMSW compared to SMS, with higher levels of essential nutrients. However, the effects of OMSW dietary supplementation on lamb meat quality parameters have not yet been examined. Therefore, the aim of this study was to investigate the effects of supplementing the diet with OMSW originating from *Pleurotus ostreatus* cultivation on lamb growth performance, meat quality, oxidative stability and fatty acid profile.

## 2. Materials and Methods

### 2.1. Lambs, Diets, and Experimental Design

The experiment was carried out in the Experimental Station of Agricultural University of Athens under natural lighting conditions (latitude 37°58′ N). Twenty-four male 90-day-old lambs of the Chios breed were used for the present study. Lambs initially consumed the same concentrate basal diet (C; Table 1) without the addition of OMSW for one week in order to become acclimatized to the experimental conditions. After this adaptation week, lambs were randomly assigned into three groups based on their body weight; one of the groups served as a control (C) and was provided with the basal diet, whereas the experimental groups were fed with the same diet supplemented with dried OMSW at 2% (P2) or 4% (P4) of DM at the concentrates. The experimental diets were formulated and manufactured at the Laboratory of Nutritional Physiology and Feeding in the Agricultural University of Athens to be isonitrogenous and isocaloric, according to the nutrient requirements for growing lambs described by NRC [15]. The duration of the main experiment was 28 days. The duration of the experimental period was considered sufficient to evaluate the primary outcome of the study, as dietary antioxidant effects on meat oxidative stability are typically evident within the timeframe of one month (±5 days), based on our previous studies [16,17,18]. The OMSW used in this study was supplied by Manitus S.A. (Athens, Greece) and consisted of stems rejected during harvesting and processing of fruiting bodies. The drying of the OMSW was carried out by the Manitus S.A. company through natural sun exposure at an ambient temperature of 25 ± 5 °C for 5 days. This process was completed when the moisture content of the material was reduced and stabilized at 12%. According to Piskov et al. [19], compared to freeze, hot air and microwave drying, solar drying enhances the antioxidant properties of the mushroom, resulting in maximal lipid oxidation inhibitory activity. Moreover, the method of drying in the sun compared to the other methods is simple, has no special requirements for expensive equipment and is characterized by significantly lower costs enabling its regular application [19]. After drying, the material was milled using an electric grinder machine (Libralon MM103, Padova, Italy) and stored in a cool (<20 °C), dry, and dark environment to limit oxidation and moisture uptake until diet formulation, which was carried out within 5 days. The proximate composition of dried OMSW (g/kg) was 12.6, 10.2, 2,09, 7.61 and 40.4 for crude protein, crude fiber, ether extract, ash and sugars, respectively, as already reported in our previous study [14].

The 3 experimental groups were housed in three different pens, each equipped with 8 individual troughs for feeding. They had the same direction and orientation and the same covered area (approximately 30 m^2^), while outdoor access was also provided. Water was ad libitum provided, while the experimental diets were administered twice daily, at 7 a.m. and 5 p.m. in individual feeders according to the treatment group (Table 1). The amount of offered feed (concentrate and forage at a mean ratio of 60:40) was calculated based on National Research Council [15]. Concentrated feed offered and remnants were recorded daily and the respective feed intake was determined by their difference. After the animals consumed the experimental concentrated diets, alfalfa hay (approximately 0.4 kg per lamb) was also provided. The conditions and facilities in each pen were checked twice per day during the experiment in order to ensure homogeneity among the treatments and avoid possible pen effects.

### 2.2. Body Weight, Carcass Traits and Internal Organ Weights

The body weight of lambs was recorded on a weekly basis in the morning before feeding (days 0, 7, 14, 21 and 28) during the experiment. At the 28th day after the beginning of the study, lambs were fasted for 12 h (water was allowed), weighed, properly anesthetized by captive-bolt stunning (European Council Directive 2010/63/EU) to induce immediate unconsciousness and slaughtered. The weights of the carcass, heart, liver, spleen, kidneys and lungs were then recorded. The carcass was then stored at 4 °C for 24 h; subsequently, the *longissimus thoracis* muscle (the 6th to 13th rib) was dissected and used for the meat quality analyses.

### 2.3. Meat Quality Analyses

#### 2.3.1. pH and Color Measurement

pH values were assessed by a pH meter (HI 99163 model, Hanna Instruments, Nușfalău, Romania) specially designed for the meat processing industry with the electrode inserted into raw meat samples. Color parameters were also determined in raw meat samples, using a Miniscan XE (HunterLab, Reston, VA, USA) chromameter, which was calibrated using a black and white tile and set on the L* (lightness), a* (redness), and b* (yellowness) systems (CIE 1976, Commission International de l’ Eclairage, Vienna, Austria).

#### 2.3.2. Cooking Loss and Shear Force Value

Meat samples (±80 g) were weighed, placed in heat-resistant plastic bags and cooked in a water bath for 35 min at 75 °C. The samples remained in the bags and were subsequently cooled under running water for 15 min and then equilibrated to room temperature [20]. They were weighed again in order to estimate cooking loss (%), which is an index of water-holding capacity. Two sub-samples with a cross section of 2 cm^2^ were cut parallel to the muscle fibers, and their shear force values were determined using a Warner–Bratzler shear blade fitted to a Zwick Testing Machine Model Z2.5/TN1S (Zwick GmbH & Co., Ulm, Germany), according to Simitzis et al. [21]. Peak force values were measured in newtons.

#### 2.3.3. Oxidative Stability

The determination of meat lipid oxidation rates was carried out by a rapid aqueous acid extraction thiobarbituric acid method for measuring MDA concentration (ng/g wet tissue) as previously described by Botsoglou et al. [22]. Malondialdehyde (MDA) values were determined on days 1, 3, 6 and 9 after storage at 4 °C; and on days 60, 120 and 180 after storage at −20 °C by a selective third order derivative spectrophotometric method (Hitachi U3010 Spectrophotometer, Hitachi, Tokyo, Japan).

#### 2.3.4. Intramuscular Fat and Fatty Acid Profile

Intramuscular fat was measured according to the method of Folch et al. [23]. Briefly, a meat sample of approximately 1 g was homogenized with a 2 chloroform:1 methanol (vol:vol) mixture to a final volume 20-fold the volume of the tissue sample. The crude extract was mixed 5:1 with distilled water and was separated into 2 phases. The lower phase contained the tissue lipids.

The meat fatty acid (FA) composition was determined by direct methylation of duplicate lyophilized samples, according to Massouras et al. [24]. In brief, the FA methyl esters (FAMES) were analyzed on a Shimadzu chromatograph (model GC-17A, Kyoto, Japan) equipped with flame ionization detector. The FAMES were separated using a SP-2560 capillary column (100 m × 0.25 mm I.D., 0.20 μm; Supelco, Bellefonte, PA, USA). The split ratio was 1:50 and helium was used as the carrier with flow rate 1.8 mL/min. The injector and detector temperatures were 250 °C and 270 °C, respectively. The injection volume was 1 μL. The initial column temperature was 45 °C (held for 3 min), then increased at 7 °C per min to 100 °C (held for 5 min) and 5 °C min to a final temperature of 220 °C (held for 20 min). The identification of the FAME peaks was performed by comparing the retention times of the FAME standards. Supelco 37 Component FAME Mix was purchased from Sigma-Aldrich (Merck KGaA, Darmstadt, Germany). A GC Solution software version 2.30 (Shimadzu Corporation, Kyoto, Japan) was used for the integration of the peaks. Individual FA content was expressed as a weight percentage (g per 100 g of total fat). The different groups of FA were determined in feed or meat as follows: Saturated fatty acids (SFA) = C10:0 + C12:0 + C14:0 + C15:0 + C16:0 + C17:0 + C18:0; Monounsaturated fatty acids (MUFA) = C15:1 + C16:1 + C17:1; Polyunsaturated fatty acids (PUFA) = C18:2n-6 + C18:3n-3 + C20:4n-6 + conjugated linoleic acid (CLA); n-3 = C18:3n-3; n-6 = C18:2n-6 + C20:4n-6; Unsaturated fatty acids (UFA) = MUFA + PUFA.

### 2.4. Statistical Analysis

Carcass traits, internal organs’ weight, meat quality characteristics, intramuscular fat and fatty acid profile were analyzed with a linear mixed model (PROC MIXED), with treatment as a fixed effect, animal ID as a random effect, and REML as the estimation method. Body weight, feed intake and MDA concentration were analyzed using a linear mixed model for repeated measurements (PROC MIXED), which included the effects of treatment and the repeated factor of sampling time and their interaction. The animal within treatment was the random effect, whereas an unstructured covariance matrix was selected based on the lowest Akaike’s Information Criterion. Body weight and weight gain were analyzed with initial body weight as a covariate. All analyses were performed using SAS/STAT [25].

## 3. Results

Feed intake was not affected by the different concentrations of OMSW incorporated into the diet. Although slight fluctuations in the values were recorded during the measurements, these were not statistically significant among the groups (*p* > 0.05, Table 2). No significant differences were also observed in the mean body weights during the 1st, 2nd, 3rd, and 4th week of the experiment (*p* > 0.05, Table 2). Moreover, no significant differences were observed in the weekly weight gain among the 3 groups (*p* > 0.05).

Final body weight and carcass weight (hot–cold) values were not significantly different among the 3 groups. No significant differences were also observed in the absolute (kg) and relative (%) weights of the internal organs (liver, lungs, heart, spleen, kidneys) (*p* > 0.05, Table 3).

As indicated in Table 4, pH_24_ did not show statistically significant differences among the groups (*p* > 0.05), and the values ranged within a narrow margin (5.74–5.76), remaining within normal limits. Regarding color, the values of the L*, a*, and b* parameters of the longissimus thoracis muscle did not also show any statistically significant difference (*p* > 0.05). Similarly, neither cooking loss (%) nor shear force (N) were affected by the incorporation of OMSW into the diet, as no statistically significant differences were recorded among the groups (*p* > 0.05). Finally, intramuscular fat values ranged from 2.10 to 2.50, with also no statistical differences observed (*p* > 0.05).

As reported in Table 5, oxidative stability during refrigerated storage (4 °C) exhibited statistically significant differences on the 3rd and 6th day (*p* = 0.01 and *p* = 0.006, respectively). In contrast, during frozen storage (−20 °C), no statistically significant differences were recorded among the groups (*p* > 0.05, Table 5). In particular, the MDA values were lower in 2% and 4% OMSW diets than control. These results suggest that the inclusion of OMSW in the diet may have contributed to the improvement of meat oxidative stability mainly during short-term refrigerated storage (4 °C).

No significant differences in the fatty acid profile of lamb meat were observed among the three groups. The addition of OMSW to the diet did not affect the concentrations of SFA, PUFA, MUFA, UFA as well as n-3, n-6 and the n-6/n-3 ratio (*p* > 0.05, Table 6), regardless of the level of mushroom inclusion in the diet. In summary, the addition of dried *Pleurotus ostreatus* at 2% and 4% levels did not affect the fatty acid profile of the lamb meat, indicating that this specific dietary intervention in the diet of the lambs does not change the lipid content and composition in the longissimus thoracis muscle.

## 4. Discussion

Nowadays, there is a constant evaluation of low-cost alternative feedstuffs that could support production goals without any side-effects [1]. In this experimental study, the effects of OMSW dietary incorporation were assessed on growth performance, carcass and meat quality traits of lambs. As indicated, the addition of OMSW neither significantly affect feed intake, body weight and weight gain during the 28 d experimental period, nor hot and cold carcass weight, and internal organ weights of lambs.

Our findings are in accordance with recent research utilizing mushroom stem waste. Mandale et al. [26] found that the provision of concentrate in which protein source was replaced by *Agaricus bisporus* waste at 10% did not negatively affect feed intake and final body weight, while simultaneously improving the body condition score of growing Berari goats. As already pointed out, studies evaluating stem waste in the nutrition of small ruminants are limited, and most of the existing literature refers to the utilization of spent mushroom substrate (SMS) and not the mushroom stem waste. However, there are significant differences in crude protein, crude fiber, ash and ether extract content between SMS [13] and mushroom stem waste [14] derived from *Pleurotus ostreatus* cultivation, highlighting the superior nutritional composition of OMSW relative to SMS.

For example, Souza et al. [27], who studied the replacement of Tifton-85 hay with dehydrated mushroom crop residue in lamb diets at levels ranging from 25% to 100%, reported that the inclusion of this spent substrate did not negatively affect growth performance, rumen pH, or carcass characteristics, as shown in our study. At the same time, Aldoori et al. [28] observed that the use of mushroom cultivation spent at a rate of up to 15% as a replacement for barley grain had no negative effect on the majority of lamb carcass characteristics. However, the same study notes that a significant decrease in the final body weight and carcass weight was observed at inclusion rates of 15–20%. These findings are in agreement with that reported by Fazaeli and Shafeyi [29], who observed a decrease in final weight as the proportion of *Agaricus bisporus* spent compost increased, a fact attributed to the decrease in organic matter and the increase of ash in the diet, resulting in a negative effect on rumen microorganisms. At the same time, Fazaeli et al. [30], in their research with mushroom spent wheat straw of *Agaricus bisporus* in feedlot calves that was incorporated into the diet in pelleted or mashed form, reported that the resulting high ash levels can affect total dry matter intake, a fact that negatively affects productive characteristics.

Furthermore, Lu et al. [31] evaluated the dietary inclusion of oyster SMS as a replacement for whole-plant corn silage in Hu sheep. Their findings indicated that a 10% substitution level was optimal, successfully enhancing nutrient digestion and absorption capacity. Conversely, higher proportions of SMS during the experimental period had a detrimental effect on feed utilization efficiency, kidney function, and blood oxygen-carrying capacity. In the same breed, Huang et al. [32] found that the addition of 15–45% fermented SMS from *Pleurotus eryngii* resulted in a significant improvement of the feed conversion ratio, as an effect of the amelioration of the rumen bacterial community structure, while meat amino acid content was increased due to the essential amino acids contained in SMS. Moreover, Koh [33] investigated the use of SMS from *Flammulina velutipes* as feed for dairy ewes. The results showed that feed intake, eating and ruminating time, and chewing activity in ewes provided with this silage tended to be greater than those in ewes fed with conventional silage, suggesting that the resultant SMS has potential for use as a roughage source.

In the present experimental study, the addition of OMSW to the lambs’ diet resulted in non-significant differences in the meat quality characteristics (pH, color, tenderness and cooking loss). Regarding literature on mushroom waste, Cvrtila Fleck et al. [34], who fed sheep a diet enriched with 1.5% dried *Agaricus bisporus* mushrooms, found statistically significant differences in meat chemical composition and color. Specifically, the meat of sheep fed with the mushroom supplement had a significantly lower percentage of fat and protein and a significantly higher percentage of water. The reduction of protein in the meat was attributed to the strong immunostimulatory effect of the white button mushroom. More specifically, a prolonged and intense immune response in the animal’s body was associated with a reduction in protein deposition in muscle tissue. Furthermore, sheep meat appeared darker, with a more intense red (higher a* value) and less yellow (lower b* value) color. The observed variation in color, compared with the findings of the present study, is likely attributable to differences in the mushroom species used.

As data on stem waste effects on meat quality remains narrow, more information can be drawn from spent substrate trials. More specifically, in a similar experiment conducted by Souza et al. [35], it was observed that the replacement of Tifton 85 hay in the sheep’s diet with *Pleurotus* spp. mushroom spent substrate at a rate of 25–100% did not alter the pH or the color parameters, namely lightness (L), redness (a), and yellowness (b), as shown in the present study, while meat sensory attributes, such as tenderness, juiciness and overall acceptance were ameliorated. The exact reasons for the observed discrepancies are speculative, but they could be attributed to different type of mushroom by-products, their supplementation level and the duration of the experimental period.

Concurrently, the oxidative stability of the meat showed statistically significant differences during refrigerated storage, whereas no statistically significant differences were presented during frozen storage. Specifically, on the 3rd and 6th day, the treatment groups exhibited significantly lower malondialdehyde levels compared to the control. Therefore, the antioxidant properties of OMSW likely have a positive effect on lamb meat oxidative stability. This finding aligns with the literature regarding the bioactive potential of mushroom residues and waste. Specifically, Aldoori et al. [28] reported that mushrooms contain many phytochemicals, such as polyphenols, which contribute to the improvement of animal health and performance. Similarly, Mandale et al. [26] highlight that mushrooms in poultry diets could eliminate free radicals owing to their antioxidant properties, further supporting the findings observed in the present study. Oyster mushrooms (*Pleurotus ostreatus*) have been shown to contain a high level of phenolic compounds, which contribute to the improvement of lipid stability in meat systems [36,37,38,39,40]. Previous research showed that mushrooms may reduce lipid oxidation, improve shelf life, and boost flavour in poultry and pork products [7].

Regarding the fatty acid profile, the results of the present study showed that the addition of OMSW neither altered the concentration of the main fatty acids, nor affected the relative abundance of saturated (SFA), monounsaturated (MUFA), and polyunsaturated (PUFA) fatty acids. Our findings can be compared to that of Cvrtila Fleck et al. [34], who fed sheep a diet enriched with 1.5% dried *Agaricus bisporus* mushrooms and found no statistically significant differences in meat fatty acid composition, recording similar values for C10:0, C12:0, C14:0, C16:0, and C18:0. Since specific data on fatty acid pathways under stem waste conditions are limited, the underlying biological mechanisms are mostly described in spent substrate literature. Furthermore, incorporating spent mushroom substrate of *Pleurotus* spp. at the expense of Tifton-85 hay into the diet of growing lambs resulted in a linear increase in t-11-C18:1 and c9t11–18:2 (CLA), while total PUFA concentrations were quadratically decreased [35]. The biological mechanism behind this lipid profile alteration is directly related to rumen physiology and changes in the digesta passage rate. Based on the biological mechanisms described by Souza et al. [35], enzymatic action on the residue can produce phenolic compounds when lignin is degraded, which can be decisive in reducing ruminal biohydrogenation, contributing to a greater flow of UFA to the intestine, with subsequent absorption and meat deposition. Due to the more than 2.5-fold lower levels (%) of crude fiber in OMSW (10.2) [14] vs. SMS (26.7) [13], these alterations in fatty acid profile were not evident in the present study.

## 5. Conclusions

The present study examined the effects of incorporating *Pleurotus ostreatus* mushroom stem waste into the diet of lambs, on production characteristics, hot and cold carcass weight, as well as internal organ weights, meat quality characteristics and oxidative stability. The evaluation of performance parameters revealed no significant effects of dietary treatment on body weight, feed intake and weight gain. Consistently, carcass traits, including hot and cold carcass weights, were not affected. In addition, meat quality attributes remained unchanged among treatments, although malondialdehyde levels were significantly lower in the treatment groups during refrigerated storage, indicating that OMSW improved meat oxidative stability possibly due to its antioxidant compounds.

In conclusion, the stem waste of the mushroom cultivation of *Pleurotus ostreatus* species can be successfully used in lamb diets up to 4%, with benefits in terms of meat oxidative stability and environmental protection within a circular economy program. As a result, OMSW can serve as a promising agro-industrial product for farm animals’ nutrition in accordance with circular economy principles, facilitating the valorization of waste streams while reducing pressure on natural resources.

## Figures and Tables

**Table 1:** Ingredients and calculated chemical composition of the diets used.

	Treatment *		
Ingredients (%)	C	P2	P4
Maize	44	44	44
Barley	13.5	13.5	13.5
Wheat bran	10	9	8
Sunflower meal 28%	8	7	6
Soyabean meal 46%	14	14.5	15
Sugar beet pulp	6	5.5	5
Calcium carbonate	1	1	1
Sodium bicarbonate	0.5	0.5	0.5
Ammonium chloride	0.5	0.5	0.5
Premix **	2.5	2.5	2.5
Oyster mushroom stem waste	0	2	4
Total	100	100	100
**Analysis (%)**			
ME (Mcal/kg) ***	2.91	2.92	2.92
Protein	16.48	16.47	16.46
Fiber	6.03	5.8	5.58
Sugars	40.07	40.51	40.95
Fat	2.62	2.61	2.6
Ash	6.67	6.69	6.71
Calcium	1.1	1.1	1.09
Total Phosphorus	0.536	0.525	0.513
Sodium	0.361	0.36	0.359
Magnesium	0.284	0.277	0.269

* C = control, P2 = 2% and P4 = 4% of OMSW in concentrated feed. ** The vitamin-mineral premix contained per kg: 21% Ca, 3.0% P, 3.0% Mg, 8.0% Na, 600,000 IU Vitamin A, 50,000 IU Vitamin D3, 1000 mg Vitamin E, along with trace elements (Zn, Mn, I, Co, Se) and B-complex vitamins. *** Metabolizable energy.

**Table 2:** The effect of dietary supplementation with oyster mushroom (*Pleurotus ostreatus*) waste on body weight and concentrate feed intake of Chios lambs (n = 8).

Average Body Weight (kg)		Treatment *			SEM	*p*-Value
		C	P2	P4		
days	0	25.37	25.26	25.50	0.55	0.951
	7	27.08	27.07	27.23	0.51	0.898
	14	28.57	28.45	28.81	0.49	0.876
	21	29.43	29.81	29.87	0.50	0.809
	28	30.06	31.11	31.41	0.59	0.256
	0–28 **	27.81	28.05	28.24	0.40	0.743
**Daily Concentrate Feed intake (kg)**						
days	0–7	0.55	0.55	0.56	0.04	0.991
	8–14	0.61	0.61	0.62	0.04	0.984
	15–21	0.63	0.64	0.64	0.04	0.979
	22–28	0.65	0.66	0.67	0.04	0.868
	0–28 **	0.59	0.60	0.61	0.04	0.981
**Weekly weight gain (kg)**						
days	0–7	1.75	1.86	1.63	0.56	0.951
	8–14	1.70	1.70	1.82	0.35	0.964
	15–21	0.87	1.36	1.05	0.25	0.386
	22–28	0.82	1.30	1.55	0.21	0.055
	0–28 **	1.11	1.37	1.45	0.15	0.256

* C= control, P2 = 2% and P4 = 4% of OMSW in concentrated feed. ** Results of the repeated measures analysis.

**Table 3:** The effect of dietary oyster mushroom (*Pleurotus ostreatus*) waste on lamb performance, carcass and internal organ weights.

Parameter	Treatment *			SEM	*p*-Value
	C	P2	P4		
Final BW, kg	28.8	30.3	30.7	1.88	0.749
Hot Carcass, kg	14.9	15.9	16.4	1.07	0.589
Cold Carcass, kg	14.3	15.3	15.8	1.05	0.645
Liver, kg	0.53	0.54	0.54	0.03	0.997
Liver, %	2.01	2.02	2.02	0.11	0.997
Lungs, kg	0.47	0.50	0.43	0.03	0.297
Lungs, %	1.79	1.88	1.64	0.11	0.297
Heart, kg	0.15	0.16	0.14	0.007	0.310
Heart, %	0.56	0.60	0.54	0.03	0.310
Spleen, kg	0.06	0.06	0.06	0.005	0.893
Spleen, %	0.21	0.21	0.22	0.02	0.893
Kidneys, kg	0.10	0.11	0.11	0.006	0.704
Kidneys, %	0.38	0.35	0.40	0.04	0.620

* C = control, P2 = 2% and P4 = 4% of OMSW in concentrated feed.

**Table 4:** The effect of dietary supplementation with oyster mushroom (*Pleurotus ostreatus*) waste on lamb meat quality (n = 8).

Parameter	Treatment *			SEM	*p*-Value
	C	P2	P4		
pH_24_	5.75	5.76	5.74	0.03	0.851
Lightness (L*)	40.4	39.9	37.5	1.01	0.117
Redness (a*)	12.8	13.0	14.1	0.50	0.162
Yellowness (b*)	15.2	14.8	15.1	0.50	0.861
Cooking loss (%)	15.4	15.8	15.1	1.25	0.934
Shear force (N)	18.9	18.8	18.56	2.18	0.992
Intramuscular fat (%)	2.10	2.50	2.10	0.27	0.500

* C = control, P2 = 2% and P4 = 4% of OMSW in concentrated feed.

**Table 5:** The effect of dietary supplementation with oyster mushroom (*Pleurotus ostreatus*) waste on oxidative stability of lamb meat during storage for 1 to 9 days at 4 °C, and for 60 to 180 days at −20 °C (ng of malondialdehyde/g of meat, n = 8).

Storage Time (Days)	Treatment *			SEM	*p*-Value
	C	P2	P4		
1	39.9	36.9	39.8	5.64	0.913
3	395.1 ^a^	340.8 ^b^	339.9 ^b^	13.1	0.010
6 at 4 °C	458.2 ^a^	410.2 ^b^	411.1 ^b^	10.6	0.006
9	648.4	589.3	575.2	33.0	0.272
1–9 **	385.4 ^a^	344.3 ^b^	341.5 ^b^	8.34	0.002
60	379.7	334.3	326.7	19.5	0.141
120 at −20 °C	531.6	491.6	512.3	15.2	0.202
180	831.6	828.3	826.5	23.2	0.988
60–180 **	581.0	551.4	555.2	10.5	0.121

* C = control, P2 = 2% and P4 = 4% of OMSW in concentrated feed. ** Results of the repeated measures analysis ^a,b^ Values sharing dissimilar superscripts within a row are significantly different.

**Table 6:** The effect of dietary supplementation with oyster mushroom (*Pleurotus ostreatus*) waste on fatty acid profile of lamb meat (n = 8).

Fatty Acid, g/100 g Fat	Treatment *			SEM	*p*-Value
	C	P2	P4		
C10:0	0.26	0.23	0.24	0.05	0.900
C12:0	0.25	0.33	0.35	0.06	0.516
C14:0	3.48	3.40	3.89	0.56	0.804
C15:0	0.21	0.27	0.27	0.05	0.585
C15:1	0.19	0.30	0.23	0.05	0.305
C16:0	25.5	23.9	25.5	0.59	0.126
C16:1	1.43	1.55	1.53	0.16	0.841
C17:0	1.04	1.16	0.98	0.07	0.186
C17:1	0.43	0.53	0.51	0.06	0.405
C18:0	17.9	16.6	15.6	0.94	0.230
C18:1n-9	36.1	35.8	37.0	1.42	0.838
C18:2n-6	6.21	7.33	5.97	0.75	0.411
C18:3n-3	0.46	0.42	0.47	0.04	0.686
CLA	0.26	0.32	0.32	0.05	0.589
C20:4n-6	1.68	2.43	2.26	0.46	0.492
SFA **	48.6	45.9	46.8	0.93	0.142
UFA	46.7	48.7	48.2	0.96	0.320
MUFA	38.1	38.2	39.2	1.48	0.842
PUFA	8.6	10.5	9.0	1.17	0.497
n-3	0.46	0.42	0.47	0.04	0.686
n-6	7.89	9.77	8.24	1.18	0.502
n-6/n-3	17.9	24.1	18.4	3.15	0.329

* C = control, P2 = 2% and P4 = 4% of OMSW in concentrated feed. ** SFA = Saturated fatty acids, UFA = Unsaturated fatty acids, MUFA = Monounsaturated fatty acids, PUFA = Polyunsaturated fatty acids. The different groups of FA were determined in feed or meat as follows: SFA = C10:0 + C12:0 + C14:0 + C15:0 + C16:0 + C17:0 + C18:0; MUFA = C15:1 + C16:1 + C17:1 + C18:1n9; PUFA = C18:2n-6 + C18:3n-3 + C20:4n-6 + CLA; n-3 = C18:3n-3; n-6 = C18:2n-6 + C20:4n-6; UFA = MUFA + PUFA.

## Data Availability

The data presented in this study are available on request from the corresponding author.

## Abbreviations

The following abbreviations are used in this manuscript:

CLA	Conjugated Linoleic Acid
MDA	Malondialdehyde
MUFA	Monounsaturated Fatty Acids
OMSW	Oyster Mushroom Stem Waste
PUFA	Polyunsaturated Fatty Acids
SFA	Saturated Fatty Acids
SMS	Spent Mushroom Substrate
UFA	Unsaturated Fatty Acids
